# Major Natural Disasters in China, 1985–2014: Occurrence and Damages

**DOI:** 10.3390/ijerph13111118

**Published:** 2016-11-10

**Authors:** Weixiao Han, Chen Liang, Baofa Jiang, Wei Ma, Ying Zhang

**Affiliations:** 1 Department of Epidemiology, School of Public Health, Shandong University, 44 West Wenhua Road, Jinan 250012, China; 201413942@mail.sdu.edu.cn (W.H.); cliang10@jhmi.edu (C.L.); bjiang@sdu.edu.cn (B.J.); 2 Climate Change and Health Center, Shandong University, 44 West Wenhua Road, Jinan 250012, China; 3 School of Public Health, University of Sydney, Sydney 2006, Australia

**Keywords:** natural disasters, spatiotemporal, changing patterns, Mann-Kendall trend test, hotspots, China

## Abstract

This study aimed to describe the characteristics of natural disasters and associated losses from 1985 to 2014. The Mann-Kendall method was used to detect any long-term trends and abrupt changes. Hotspot analysis was conducted to detect the spatial clusters of disasters. We found an increasing trend in the occurrence of integrated natural disasters (*tau* = *0.594*, *p* < 0.001*),* particularly for floods (*tau* = 0.507, *p* < 0.001), landslides (*tau* = 0.365, *p* = 0.009) and storms (*tau* = 0.289, *p* = 0.032). Besides, there was an abrupt increase of natural disasters in 1998–2000. Hotspots of droughts, floods, landslides and storms were identified in central, southern, southwest and southeast areas of China, respectively. Annual deaths from integrated natural disasters were decreasing *(tau* = −0.237, *p* = 0.068) at about 32 persons/year, decreasing at 17 persons/year for floods (*tau* = −0.154, *p* = 0.239), and decreasing at approximately 12 persons/year for storms (*tau* = −0.338, *p* = 0.009). No significant trend was detected in inflation-adjusted damages while a declining trend was detected in the ratio of year damage against GDP (gross domestic product). In conclusion, there has been an increasing trend in occurrence of natural disasters in China with the absence of an increase in life and economic losses. Despite the progress in the disaster adaption, there will be great challenges in disaster control for China in the future.

## 1. Introduction

Natural disasters are threats to populations because they could derail socioeconomic development, strain social safety nets, and usually require comprehensive emergency responses. People are vulnerable to natural disasters, particularly in developing countries, where the availability of disaster relief resources is limited [1,2]. During the last decade alone, Indonesia, Sri Lanka, Pakistan, China, Haiti, and Japan have all experienced natural disasters with high death tolls [3]. Droughts, wildfires, floods, and extreme weather events have led to serious economic losses [4]. Such disasters may occur more frequently [5]. However, various component features of the climatic extremes, e.g., the dominant disaster type and the economic loss and death burden demonstrated mixed and inconclusive trends with considerable geographical variations [6].

China is one of the countries that suffer from frequent disasters because of the vast territory, various climatic zones, complex geographical environment, and fragile ecological conditions [7]. During the past three decades, China has experienced great changes in its population size, development patterns, economic conditions, and socio-economic status. Therefore, it is important to understand the distribution and patterns of natural disasters, as well as life and economic losses, for better disaster responses in China. This study aims to analyze the temporal and spatial distribution of natural disasters in China, including their frequencies and impacts.

## 2. Materials and Methods

### 2.1. Data Collections

We obtained the disaster data from the Emergency Events Database (EM-DAT). EM-DAT is operated by the Center for Research on the Epidemiology of Disasters (CRED) at Louvain University in Ottignies-Louvain-la-Neuve, Belgium. It contains essential core data on the occurrence and effects of over 22,000 mass disasters in the world from 1900 to the present day. The database is compiled from various sources, including UN agencies, non-governmental organizations, insurance companies, research institutes and press agencies. Loss data from EM-DAT are mainly based on information from UN agencies, government offices, International Federation of Red Cross and Red Crescent Societies (IFRC), research organizations, insurance periodicals (Lloyd’s List), and reinsurance publications [8,9]. For a disaster to be included into the database, at least one of the following criteria must be fulfilled: (1) ten or more deaths were reported; (2) one hundred or more people were affected; (3) declared a state of emergency; of (4) called for international assistance [9]. The data contained the following information for each disaster: country, disaster group, disaster type, location, date, number of affected people, duration of each disaster, and estimated disaster-related economic loss. Fundamental digital maps of China were obtained from the National Earth System Science Data Sharing Infrastructure [10].

Data for gross domestic product (GDP) in dollars were obtained from the Word Economic Information Network [11]; data for the consumer price index (CPI) were obtained from the National Bureau of Statistics of the People’s Republic of China [12].

### 2.2. Classifications and Definitions

Disasters were divided into six types: droughts, wildfires, floods, landslides, extreme temperature events (ETEs), and storms. Wildfires included land fires and forest fires. Floods included coastal floods, flash floods, and riverine floods. Avalanches were classified as landslides. Extreme temperature events consisted of heatwaves, cold waves, and severe winter conditions. Storms included convective storms and tropical cyclones. The integrated natural disasters below in this article refer to the major disaster types, including droughts, wildfires, floods, landslides, ETEs, and storms. The estimated economic loss in EM-DAT included the damage of property, crops, and livestock. For each disaster, estimated losses in the database corresponded to the value at the moment of the event [9].

### 2.3. Statistic Analysis

In this study, we used the data from 1985–2014 to detect any trends and patterns in the frequency of natural disasters, as well as death tolls and economic losses. Data from Hong Kong, Macao, and Taiwan were excluded, in view of the differences in disaster policies, strategies, and other development progress in these regions from those in mainland China. Geographical variability was also described in this study. The Mann-Kendall method was used to conduct trend tests and we calculated the Kendall’s tau using the “Kendall” package in R 3.1.3 (R Foundation for Statistical Computing, Vienna, Austria). The Kendall’s tau has the attractive property that it lies on a scale from −1 to 1, where values of 1, −1, and 0 signify a perfect increasing trend, a perfect declining trend, and no overall trend [13,14]. Sen-slopes were calculated to assess the degree of change. We further detected the abrupt change point (ACP) based on the Mann-Kendall method combined with T tests. The details of the computation of Sen-slopes and ACP detection can be found in Supplementary Materials Section 1.

Hotspot analysis was conducted to detect spatial clusters of the disasters. The hotspot analysis calculated the Getis-OrdGi* statistics for each disaster frequency of all the studied provinces. This method works by looking at each frequency within the context of neighboring frequencies. The z-scores and *p*-values indicate spatially-clustered areas with frequencies of either high or low values. For statistically significant positive z-scores, the larger the z-score, the more intense the cluster of high values (hot spots). For statistically significant negative z-scores, the smaller the z-score, the more intense the cluster of low values (cold spots). We have tried different settings of distance thresholds in the analysis. First, we retained the default settings in ArcGIS software (Environmental Systems Research Institute, Redlands, CA, USA), which ensured each unit has at least one neighbor. However, for the hotpot analysis, the default choice of the distance threshold was not always the most appropriate. Instead, the radius that ensures eight neighbors for each unit was considered the most reasonable. Therefore, we further set the average distance among every eight neighboring units as the distance threshold in the analysis. We found the latter results were in line with the former results.

For economic loss detection, in order to ensure inter-annual comparability, we used the record value of yearly total damage (TD) and the corresponding consumer price indexes (CPIs) to compute the CPI-adjusted damage (CPIAD), and then further examined the long-term trend. The employed CPI on a certain year over the studied period was the CPI of 2014 calculated based on that year. We computed the CPI-adjusted damage based on the following equation: CPI-adjusted damage=TD × CPI × 0.01. Additionally, TD/GDPs were also calculated to reflect the trend in economic losses to reduce the bias of the detected result based on a single index. For life loss detection, annual deaths and annual mortality were calculated to examine the trend.

In this study, we did not conduct the trend analysis for disaster types with low frequencies (droughts, wildfires, and ETEs), but the spatial distribution of these three disaster types was also of interest and involved in this study. 

### 2.4. Ethical Approval

A review by the Institutional Review Board was exempted by the Ethics Review Committee (ERC#: 20150115) at the School of Public Health, Shandong University, because no individual data of human subjects was involved in this study.

## 3. Results

### 3.1. Occurrence of Natural Disasters

**Figure 1:**
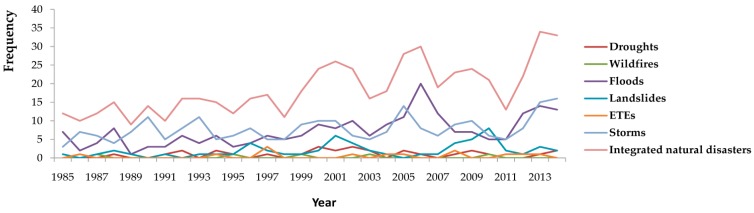
Occurrence of natural disasters in China, 1985–2014.

In total, 558 natural disasters occurred in China over the study period, including 30 droughts, five wildfires, 216 floods, 59 landslides, 13 ETEs, and 235 storms. Time series of the frequencies of different disasters are shown in Figure 1. The average frequencies of droughts, wildfires, floods, landslides, ETEs, and storms were: 1.0, 0.2, 7.2, 2.0, 0.4, and 7.8 per year, respectively. Storms and floods were the most frequent disasters. In terms of seasonal patterns, droughts occurred mostly in January and June, while most wildfires occurred in April. Floods and landslides often occurred in warm seasons from June to August, and seldom occurred in cold seasons from November to the following April. Storms, however, occurred most frequently in warm seasons, from July to September.

### 3.2. Temporal Trend of the Natural Disasters

There was a significantly increasing trend in the occurrence of integrated natural disasters (*tau* = 0.594, *p* < 0.001) from 1985–2014. Among the six types of disasters, there was a significant increasing trend in the occurrence of floods (*tau* = 0.507, *p* < 0.001), landslides (*tau* = 0.365, *p* = 0.009), and storms (*tau* = 0.289, *p* = 0.032). The frequency of integrated natural disasters increased at 0.57 per year, among which floods contributed the most. The frequency of floods increased at 0.30 per year, while that of storms increased at 0.14 per year (Table 1). In terms of affected areas, number of flood episodes which affected a single province was 105, accounting for 48.6% of the total floods. Flood episodes attacking multiple provinces were dominant in the period of 1990–1994, 1995–1999, and 2010–2014. The number of storm episodes which affected a single province was 119, accounting for 50.6% of the total storms. However, the constituent ratio of storm episodes attacking multiple provinces was increasing with time. The details are shown in Table 2.

**Figure 2:**
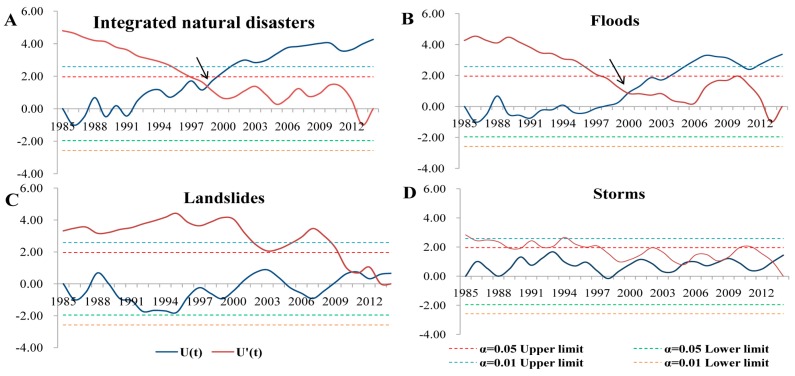
Abrupt change analysis of natural disasters in China, 1985–2014 (based on the Mann-Kendall method). (**A**) Integrated natural disasters; (**B**) floods; (**C**) landslides; and (**D**) storms.

We further detected the abrupt change points (ACPs) of natural disasters. In Figure 2, the joint points of two lines were the possible ACPs. For integrated natural disasters, the possible ACP was around 2000. The possible ACPs for floods, landslides, and storms were around 2000, 2012, and 2005, 2008, 2013 respectively. According to the results of the *t*-test, 2000 was indeed a booming point for the number of integrated natural disasters (*t* = 2.85, *p* = 0.022) and floods (*t* = 4.02, *p* = 0.004). However, for floods, a significant increase already started from 1998 (*t* = 2.72, *p* = 0.026).

### 3.3. Spatial Patterns of the Natural Disasters

**Figure 3:**
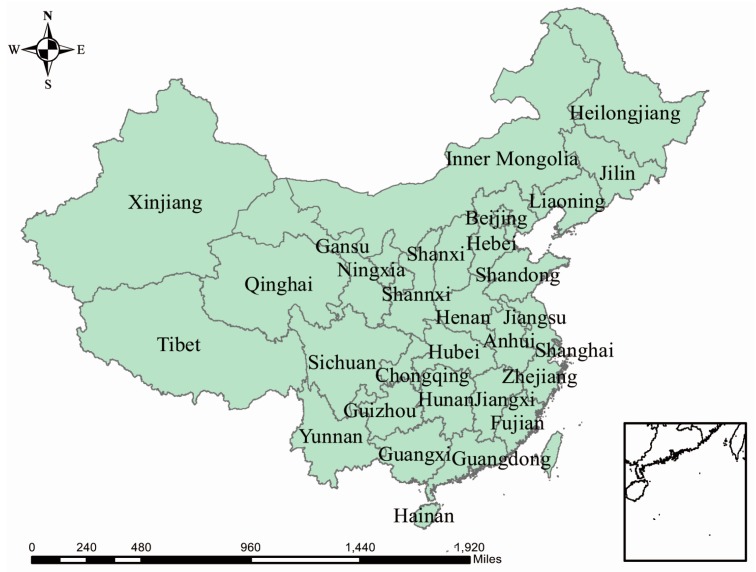
A map of Greater China labeled with the names of analyzed provinces.

**Figure 4:**
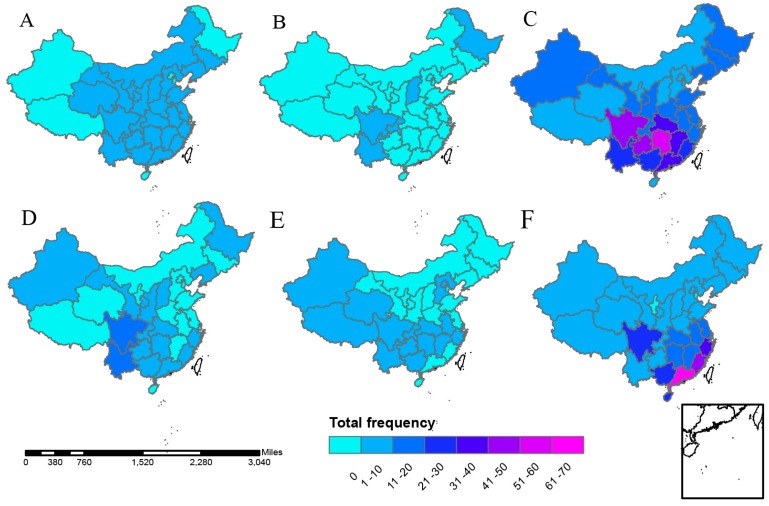
The spatial patterns of natural disasters by types in China, 1985–2014. (**A**) Droughts; (**B**) wildfires; (**C**) floods; (**D**) landslides; (**E**) ETEs; and (**F**) storms.

The names of the analyzed provinces are listed in Figure 3. Figure 4 shows the spatial distribution of different natural disasters. The provinces suffered most frequently from droughts were: Shandong, Anhui, Hunan, Inner Mongolia, and Sichuan, with 5–6 droughts occurring in total. Wildfires seldom occurred, with most of them occurring in Heilongjiang (Northeast China). As the most affected provinces, Sichuan, Hunan, and Guangxi had been flooded more than 41 times. South inland, Beijing and Shanghai were relatively more affected than others by ETEs. Southern coastal provinces suffered frequently from storms, among which Guangdong ranked the first with 68 storms occurring in total.

**Figure 5:**
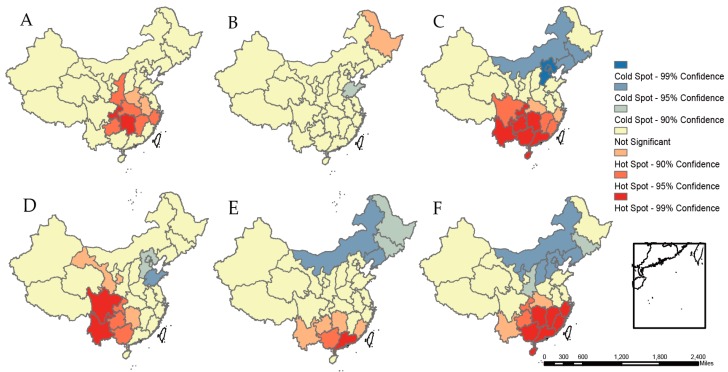
Hotspot analysis of natural disasters by types in China, 1985–2014. (**A**) Droughts; (**B**) wildfires; (**C**) floods; (**D**) landslides; (**E**) ETEs; and (**F**) storms.

The results of hotspot analysis [15] are visualized in Figure 5. For droughts, at a significance level of 1%, hotspots were Chongqing municipality and Hunan province, while at a significance level of 5%, hotspots expanded to Shanxi, Hubei, Jiangxi, Zhejiang, Guizhou. At a significance level of 1%, hotspots of floods covered Yunnan, Guizhou, Chongqing, Hunan, Guangdong, Hainan, and Guangxi, located in the southwest. At a significance level of 1%, hotspots of landslides were Sichuan and Yunnan province, basically in Southwest China, while at a significance level of 5%, hotspots expanded to neighboring Chongqing, Guizhou, and Guangxi. With regard to storms, hotspots at the significance level of 1% consisted of Hunan, Jiangxi, Zhejiang, Fujian, Guangdong and Zhejiang. No significant hotspots were detected for wildfires or ETEs.

### 3.4. Deaths in Natural Disasters

**Figure 6:**
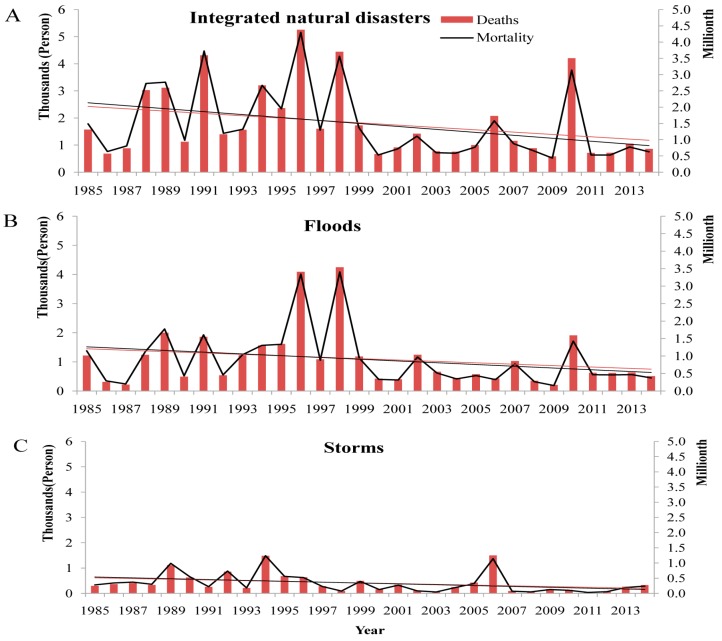
Life losses caused by integrated natural disasters, floods, and storms in China, 1985–2014. (**A**) Integrated natural disasters; (**B**) floods; and (**C**) storms. Note: the *y*-axes on the left correspond to the indexes of “Deaths”; the *y*-axes on the right correspond to the index of “Mortality”; the line in red denotes the changing trend of deaths, while the line in black denotes that of mortality.

In total, natural disasters led to 54,086 deaths over the study period. Among these deaths, 3534 (6.5%) were caused by droughts, 265 (0.5%) by wildfires, 33,007 (61.0%) by floods, 4713 (8.7%) by landslides, 379 (0.7%) by ETEs, and 12,188 (22.5%) by storms. The annual number of deaths and mortality from integrated natural disasters, floods, and storms are presented in Figure 6. Deaths from integrated natural disasters decreased at about 32 persons per year, but no significant trend was observed (*tau* = −0.237, *p* = 0.068). Deaths from floods were decreasing at 17 persons per year, and no trend was observed (*tau* = −0.154, *p* = 0.239). However, we detected a decreasing trend in annual deaths caused by storms (*tau* = −0.338, *p* = 0.009) at approximately 12 persons per year. The trend of mortality from natural disasters has a similar pattern with deaths (Table 1). The statistic of deaths from natural disasters during sub-periods was shown in Supplementary Materials Table S1.

### 3.5. Economic Losses Caused by Natural Disasters

**Figure 7:**
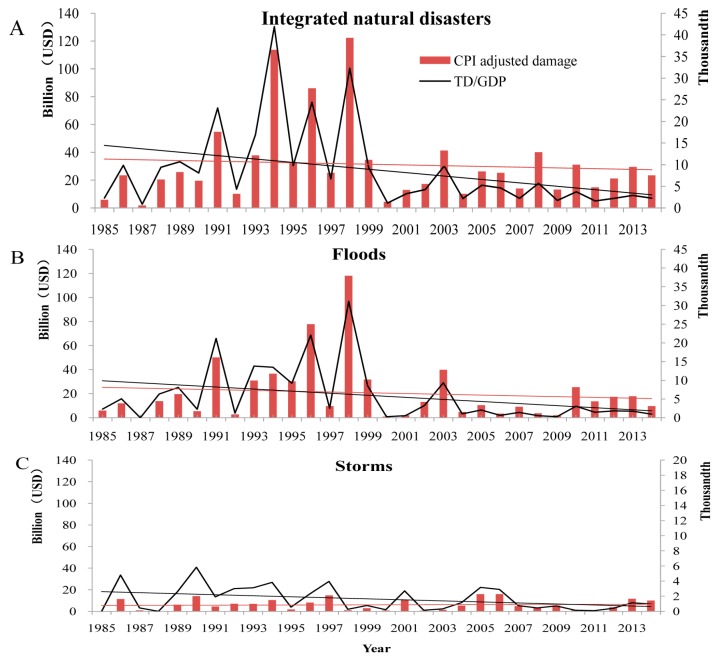
Economic losses caused by integrated natural disasters, floods, and storms in China, 1985–2014. (**A**) Integrated natural disasters; (**B**) flood; and (**C**) storms. Note: The *y*-axes on the left correspond to the index of “CPI-adjusted damage”; the *y*-axes on the right correspond to the indexes of “TD/GDP”; the line in red represents the changing trend of CPI-adjusted damage, while the line in black represents that of TD/GDP.

From 1985–2014, economic losses from natural disasters were estimated at US$874,613 million in total in 2014 dollars. Among these, US$35,397 million (4.0%) were caused by droughts, US$831 million (less than 0.1%) were caused by wildfires, US$618,433 million (70.7%) were caused by floods, US$4,772 million (0.5%) were caused by landslides, US$33,312 million (3.8%) were caused by ETEs, and US$181,868 million (20.8%) were caused by storms. Economic losses from integrated natural disasters, floods, and storms are presented in Figure 7. The CPIAD and TD/GDP were included. No significant trend was detected in CPIAD, while a declining trend was detected in the TD/GDP (Table 1).

## 4. Discussion

Generally, China has suffered from increasing natural disasters in the last three decades. However, the number of deaths has generally declined and the property losses remained stable from natural disasters. This may be explained by the progress of disaster adaption in China [16]. Jia et al.’s study based on the Chinese provincial newspaper database of natural disasters also reported increasing disasters in China and their extending affected area [17]. Similarly, Qin et al. found that since the 1980s, the deaths toll in weather- or climate-related disasters declined steadily [18].

We have found floods and storms have been the most frequent natural disasters in China. We have also detected a significant increasing trend in the frequencies of floods and storms. The size of the affected area extended beyond a single province in a single natural episode. However, the annual deaths from floods did not present any statistically significant trend, while the deaths from storms were significantly decreasing. At the same time, neither property losses from floods nor that from storms showed any significant trend. The finding of increasing storms is in accordance with Wu et al.’s prediction that tropical cyclones will increase with the ongoing warming climate [19].

Flooding is the overflowing or failing of the normal confines of a river, stream, lake, sea, or accumulation of water as a result of heavy precipitation through lack, or exceeding of, the discharge capacity of drains [20,21]. Due to its geographic position, topography, and climate, China often faces floods [22]. A high amount of average rainfall and volume of river water in China made the changes in the hydrological conditions and flood risks had been the especially focusing issues [23]. Additionally, storms accompanied with heavy precipitation also likely lead to floods. 

In terms of disaster frequency activities, such as population growth, deforestation, urbanization, and industrialization, have accelerated environmental degradation (e.g., climate variability and sea level rise), and increased the potential occurrence of disasters, particularly floods in consequence [5,24]. According to National Bureau of Statistics [12], China experienced a noticeable shift in population: In 1985, China’s population was approximately 1.0 billion, while the urban population accounted for 23.7%. In contrast, by the end of 2014, China had a total population of 1.3 billion, while the urban population accounted for 54.8%. This massive population growth and urbanization has significant natural environment and public health implications. The expansion of human settlements and accompanying activities that occurred in China may cause changes to ecological processes, and increase the disaster frequency, as well as extend the affected area in a single disaster episode. Additionally, the detected ACPs in 1998–2000 are in line with the widely-recorded anomalous flood episodes in 1998, which was an El Niño year [25,26,27]. This detected ACP in 1998 might verify the data reliability for the trend analysis to some degree. It is worth mentioning that many of the climate anomalies associated with the Pacific Decadal Oscillation (PDO) are broadly similar to those connected with ENSO variations (EI Niño and La Niña), though generally not extreme. Mantua et al. reported that, in the last two decades of the 20th century, the extra-tropical Pacific Ocean was in an almost continuous EI Niño-like state despite the absence of tropical EI Niño events in a majority of those years [28]. Similarly, Webster et al. found that recent increases of sea surface temperature (SST) seem to be responsible for the increased intensity tropical cyclones over the past 35 years [29,30]. Thus, the Pacific inter-annual variability may also play a role in the increasing trend of disasters, which should be explored in more in-depth studies in the future.

In terms of losses, population density and magnitude, land use, warning and emergency systems will have influences on the loss from natural disasters. However, the level of disaster protection and the organization of disaster defense and management are also important factors [22,31]. In the 1990s, especially after the 1998 flood, China undertook strategic adaptation and mitigation in responding to floods. The focus of flood prevention has shifted from flood control to flood management. Since 2000, China’s natural disaster emergency management system has been in a fundamental reform process [32,33]. The Ministry of Civil Affairs National Disaster Reduction Center was established and the rescue plan was further improved (31 provincial natural disaster rescue contingency plans were developed, 310 cities and 2347 counties have developed disaster relief contingency plans, and the national and the disaster relief emergency response system was initially established) [34,35]. In short, the Chinese government has strengthened the development of disaster management, and established a national management system with Chinese characteristics related to disaster prevention and reduction [18,36]. Thus, in China, communities might be more resilient to major natural disasters, such as floods, than developed countries due to experience with past major disasters and a strong social, structural, and coping capacity. However, compared with developed countries, China still has fewer resource investments for sustainable protection strategies [37]. This may be explained by the absence of a certain trend in economic loss in the context of the increasing disasters. Additionally, with regard to the stable loss from floods and the significant decreasing deaths from storms, economic growth may be of great relevance. Economic status of societies is determined to a large extent their capability to cope and “live” with disasters [20,38].

China’s southeast coastal areas constitute the storm-prone zone, under the influence of tropical cyclones generated in the Western North Pacific. Wei et al. have found that Southeastern China was at a higher risk of multiple meteorological hazards as a result of their geographical location and topography [39]. This is in line with our findings in the geographical distribution of storm episodes. 

As for landslides, a significant increasing frequency has also been detected. However, it was not accompanied by an increase in deaths or economic losses. This general trend condition was similar with that of floods. On one hand, rainfall and seismicity are the main natural triggers of landslides [40,41]. In China, almost 75% of the total land area is mountainous. Meanwhile, the rainfall distribution is extremely uneven under a monsoon climate. On the other hand, in densely-populated regions, the impacts of humans on the environment contribute significantly to the initiation and reactivation of landslides [40,42,43,44,45,46]. Thus, landslide episodes usually are serious in developing countries where the environment protection and management are hardly sustainable [43,45,47]. We have found that landslide episodes might significantly concentrate in Southwest China, which is in line with previous studies [48,49]. The spatial patterns of landslide occurrence maybe largely due to the topography, geology, and rainfall distribution. In China, the terrain is high in the west and low in the east. The western land is mainly covered with vast plateaus and towering mountains, and the eastern land mainly consists of undulating hills and flat plains. Development in southwest expands into unstable hill slope areas under the pressures of increasing population and urbanization could make this area vulnerable. Meanwhile, heavy rainfall is more frequent in the south than the north, with its concentration in summers causing most landslides to be concentrated from June to August. Life losses from landslides in China have always been a serious problem. It is reported that more than 110 people per year on average were killed by landslides from 1951–1987 [48]. In this study, we could not detect any trend in deaths due to landslides. 

Droughts, wildfires, and ETEs had a very low frequency, as detected in our study. We believe that there are two main possible reasons. First, the frequency of these disasters is low in itself. Second, these disasters were difficult to be identified and failed to be recorded because of their development characteristics and influence degrees. For instance, droughts are not recognized as natural disasters unless human activity is affected. However, unlike most other natural disasters, droughts usually develop slowly and their onset is generally difficult to detect. Direct and immediate death caused by drought is seldom [50], whereas the duration of droughts can be several weeks, months, and even years [9,51]. Furthermore, droughts can also have disastrous consequences to local environmental and socio-economic conditions [52]. According to Supplementary Materials Table S2 in our study, the average annual economic loss from droughts closely followed the flood episodes. Additionally, hotspots of droughts may roughly located in provinces along the Yellow River and the Yangtze River basin. In the literature, drought is often considered the most complex natural disaster with more people affected than by any other disasters [53,54,55]. It is not solely a physical phenomenon because its impacts can be exacerbated by human activities and water supply demands [52,54,56]. In short, these disasters with a low frequency cannot be ignored and their impact assessment needs to be discussed and improved in further studies.

## 5. Limitations

First, this study solely relied on the EM-DAT as a single source for analysis. The EM-DAT is the only publicly accessible loss data source among the current existing global loss databases (NatCatSERVICE, Sigma, and EM-DAT). However, among the three global databases, the inclusion criteria of EM-DAT for record disasters were stricter. Additionally, it is widely employed in reports and studies in various geographical and temporal scopes. Guha-Sapir et al. have compared publicly accessible EM-DAT with two private disaster databases, including NatCatSERVICE and Sigma. Differences exist between databases with respect to the number of events included and completeness of records. However, due to the increasing commonality of data sources these differences reduced significantly with time, e.g., records that date from the 1990s had smaller discrepancies than those from the 1980s [8]. Furthermore, in the abrupt change point (ACP) detection, we detected the ACP of 1998, an El Niño year when China experienced abnormal flood episodes, recorded as well in other literature [25,26,27]. Thus, we believe the trend detection was reliable based on the current source.

Second, in this study, the severity degree of a natural disaster was not involved in our analysis due to data unavailability. However, we conducted an exploratory analysis showing the number of affected provinces in a single disaster episode, which we believe may be make up for this shortcoming to some extent.

Third, the significant increasing trend has not been adjusted by several other factors (e.g., the growth in exposure) which could have influence on the occurrence of natural disasters. What we have achieved in this study is the description of general conditions of major natural disasters in China within the context that many changes would occur over the long-term study period. Further investigations could be conducted with more available data.

## 6. Conclusions

There has been an increasing trend in the occurrence of natural disasters in China in the last three decades with the absence of an increase in life and economic losses. Despite the progress in the disaster adaption, there will be great challenges in disaster control for China in the future. Given the geographic variation in hotspots of the disasters, it would be critical and challenging to develop a national policy and strategies to prevent associated losses in the future.

## Figures and Tables

**Table 1:** Mann-Kendall trend test with the Sen-slope of natural disaster occurrence and the resulting deaths and damages in China, 1985–2014.

Disasters	Frequency			Life loss						Damages					
				Deaths			Mortality			CPIAD			TD/GDP		
	*tau*	*p*	*Sen*	*tau*	*p*	*Sen*	*tau*	*p*	*Sen* *	*tau*	*p*	*Sen* **	*tau*	*p*	*Sen* ***
Integrated	0.594	<0.001	0.57	−0.237	0.068	−32.12	−0.313	0.017	0.03	0.039	0.775	133.98	−0.338	0.009	−0.27
Floods	0.507	<0.001	0.30	−0.154	0.239	−17.00	−0.206	0.126	−0.02	−0.025	0.858	−47.21	−0.240	0.066	−0.13
Landslides	0.365	0.009	0.06	0.056	0.681	0.65	0.035	0.802	0	-	-	-	-	-	-
Storms	0.289	0.032	0.14	−0.338	0.009	−11.67	−0.406	0.003	−0.01	0.078	0.556	43.37	−0.160	0.224	−0.05

Note: * the Sen-slope of mortality was calculated in the unit of millionths; ** the Sen-slope of CPIAD (CPI-adjusted damages) was calculated in the unit of million US dollars; *** the Sen-slope of TD/GDP (Total damages/Data of Gross Domestic Product) was calculated in the unit of thousandths; we have not tested the trend of damages from landslides because there are too many zeroes in the time series.

**Table 2:** The shift in number of affected provinces in a single natural disaster in China, 1985–2014.

Disasters	NumberAP	1985–1989	1990–1994	1995–1999	2000–2004	2005–2009	2010–2014	Total
		Es(%)	Es(%)	Es(%)	Es(%)	Es(%)	Es(%)	Es(%)
Floods	1	13(59.1)	10(45.5)	9(37.5)	26(61.9)	36(62.1)	11(22.4)	105(48.6)
	2–5	7(31.8)	7(31.8)	9(37.5)	11(26.2)	17(29.3)	24(49.0)	75(34.7)
	6–9	1(4.5)	3(13.6)	3(12.5)	2(4.8)	3(5.2)	8(16.3)	20(9.3)
	10~	0(0)	0(0)	2(8.3)	2(4.8)	1(1.7)	2(4.1)	7(3.2)
	Uncertain	1(4.5)	1(4.5)	1(4.2)	1(2.4)	0(0)	4(8.2)	8(3.7)
	Total	22(100)	22(100)	24(100)	42(100)	57(100)	49(100)	216(100)
Storms	1	19(70.4)	26(66.7)	20(60.6)	19(50.0)	21(44.7)	14(28.0)	119(50.6)
	2–5	7(25.9)	13(33.3)	11(33.3)	16(42.1)	20(42.6)	24(48.0)	91(38.7)
	6–9	0(0)	0(0)	0(0)	0(0)	3(6.4)	3(6.0)	6(2.6)
	10~	0(0)	0(0)	0(0)	0(0)	0(0)	0(0)	0(0)
	Uncertain	1(3.7)	1(2.6)	2(6.1)	3(7.9)	3(6.4)	9(18.0)	19(8.1)
	Total	27(100)	40(100)	33(100)	38(100)	47(100)	50(100)	235(100)

Note: NumberAP denotes the number of affected provinces; Es denotes the frequency of disaster episodes.

## Supplementary Materials

The following are available online at http://www.mdpi.com/1660-4601/13/11/1118/s1, Table S1: Deaths caused by different natural disasters in China, 1985–2014; Table S2: Damages (Million USD in 2014 price) from different natural disasters in China, 1985–2014.
