# Supplementary material for: Major Natural Disasters in China, 1985–2014: Occurrence and Damages
Source: Int J Environ Res Public Health. 2016 Nov 10;13(11):1118. doi: 10.3390/ijerph13111118 (PMC5129328; doi:10.3390/ijerph13111118)
Supplement: Supplementary file 1 [file ijerph-13-01118-s001.pdf]

Supplementary Materials: Major Natural Disasters in China, 1985–2014: Occurrence and Damages

Weixiao Han, Chen Liang, Baofa Jiang, Wei Ma and Ying Zhang

1. Description of Statistical Analysis

1.1. Mann-Kendall Trend Test

The Mann-Kendall test has been widely used to test for randomness in hydrology and climatology [1]. The Mann−Kendall test [1–4] statistics is calculated via the following equation:

$$S=\sum_{i=1}^{n-1}\sum_{j=i+1}^{n}\mathrm{sgn}(x_j - x_i) \tag{1}$$

where n is the number of data points, x_i and x_j are, respectively, the i_{th} and j_{th} data values in the data sequence ($j > i$). The $\mathrm{sgn}(x_j - x_i)$ is a sign function determined as follows:

$$\mathrm{sgn}\left(x_j - x_i\right)=\begin{cases} +1, \text{if } (x_j - x_i) > 0 \\ 0, \quad \text{if } \left(x_j - x_i\right) = 0 \\ -1, \text{if } \left(x_j - x_i\right) < 0 \end{cases} \tag{2}$$

In given conditions where the sample size $n > 10$, the mean ($\mu(S)$) and variance ($\sigma^2(S)$) are given as:

$$\mu(S)=0 \tag{3}$$

$$\sigma 2(S)=\frac{n(n-1)(2n+5)}{18} \tag{4}$$

The standard normal test statistic Z_S is calculated as:

$$Z_S=\begin{cases} \frac{S-1}{\sqrt{\sigma^2(S)}}, & \text{if } S > 0 \\ 0, & \text{if } S = 0 \\ \frac{S+1}{\sqrt{\sigma^2(S)}}, & \text{if } S < 0 \end{cases} \tag{5}$$

The significance level of $\alpha = 0.05$ was used in this study. A positive value of Z_S indicates an increasing trend, whereas negative indicates decreasing trend. At the given significance level, the null hypothesis of no existing trend is rejected if $|Z_S| > 1.96$.

1.2. Abrupt Change Point Detection

Abrupt change analysis is based on the sequential Mann-Kendall test. Sequential values of U(t) and U′(t) from the analysis of the Mann-Kendall test were determined in order to detect the change of the trend over time. U(t) is a standardized variable that has zero mean and unit standard deviation. U(t) is the same as the Z values that are found from the first to last data point. The steps applied in sequence are as follows:

The magnitudes of x_j time series (j=1, 2, 3, …, n) are compared with x_i (i = 1, 2, 3, …, j−1). At each comparison, the number of cases $x_j > x_i$ is counted and denoted by n_j.

The test statistic t is:

$$t_j = \sum_{1}^{j} n_j \tag{6}$$

The mean and variance of the test statistic are:

$$E_t = \frac{n(n-1)}{4} \tag{7}$$

The sequential values of the statistic U(t) are:

$$\mathrm{U}(t) = \frac{t_j - \mathrm{E_t}}{\sqrt{\mathrm{Var}(t_j)}} \quad (8)$$

Similarly, the values of U′(t) are computed backward, starting from the end of the time series. The sequential version of the Mann–Kendall could be considered as an effective way of determining the possible starting point of a trend. We draw the U(t) and U′(t) in the same figure and consider the join point of the two lines as the possible abrupt change point. Then, the t test was used to verify this change point. We compare the average level of values of five years before and after this point. If the null hypothesis of no difference is rejected, then we consider this point as a true abrupt change point.

1.3. Computation of Sen-Slope

The slope estimated by using a non-parametric procedure developed by Sen can indicate the degree of change per unit time [5]. The slope estimates of N pairs of data are first computed by:

$$Q_i = \frac{x_j - x_i}{j - i} \quad \text{for } i = 1, 2, 3, \ldots, N \quad (9)$$

The median of these N values of Q_i is Sen's estimator of slope. If N is odd, then Sen's estimator is computed by $Q_{\mathbf{med}} = Q_{(N+1)/2}$.

Table S1. Deaths caused by different natural disasters in China, 1985–2014.

Year	Droughts	Wildfires	Floods	Landslides	ETEs *	Storms
1985–1989	1400	191	4991	181	2	2520
1990–1994	2000	0	5691	368	104	3444
1995–1999	0	52	12,234	880	40	2192
2000–2004	0	0	3182	389	46	924
2005–2009	134	0	2599	570	145	2256
2010–2014	0	22	4310	2325	42	852
Total	3534	265	33,007	4713	379	12,188
Average/Per Year	118	9	1100	157	13	406

* Extreme temperature events.

Table S2. Damages (Million USD in 2014 price) from different natural disasters in China, 1985–2014.

Year	Droughts	Wildfires	Floods	Landslides	ETEs *	Storms
1985–1989	6607	831	51,254	0	0	18,549
1990–1994	13,808	0	125,890	0	0	42,965
1995–1999	182	0	267,525	3310	114	28,678
2000–2004	2006	0	60,926	186	0	18,854
2005–2009	6744	0	28,756	211	32,817	45,259
2010–2014	6050	0	84,082	1065	381	27,563
Total	35,397	831	618,433	4772	33,312	181,868
Average/Per Year	1180	28	20,614	159	1110	6062

* Extreme temperature events.

References

1. Partal, T.; Kahya, E. Trend analysis in Turkish precipitation data. *Hydrol. Process.* **2006**, *20*, 2011–2026.
2. Zhao, W.; Yu, X.; Ma, H.; Zhu, Q.; Zhang, Y.; Qin, W.; Ai, N.; Wang, Y. Analysis of precipitation characteristics during 1957–2012 in the semi-arid Loess Plateau, China. *PLoS ONE* **2015**, *10*, e0141662.
3. Yue, S.; Wang, M.C. The mann-kendall test modified by effective sample size to detect trend in serially correlated hydrological series. *Water Resour. Manag.* **2004**, *18*, 201–218.
4. Wang, L.; Shao, Q.; Chen, X.; Li, Y.; Wang, D. Flood changes during the past 50 years in Wujiang River, South China. *Hydrol. Process.* **2012**, *26*, 3561–3569.
5. Sen, P.K. Estimates of the regression coefficient based on Kendall's tau. *J. Am. Stat. Assoc.* **1968**, *63*, 1379–1389.
